# Reduction chemistry of neptunium cyclopentadienide complexes: from structure to understanding†
†Electronic supplementary information (ESI) available: General procedures, synthetic details, spectroscopic data, X-ray crystallographic data. CCDC 1524162–1524166. For ESI and crystallographic data in CIF or other electronic format see DOI: 10.1039/c7sc00034k



**DOI:** 10.1039/c7sc00034k

**Published:** 2017-01-30

**Authors:** Michał S. Dutkiewicz, Christos Apostolidis, Olaf Walter, Polly L. Arnold

**Affiliations:** a European Commission , Directorate for Nuclear Safety and Security , Joint Research Centre , Postfach 2340 , D-76125 , Karlsruhe , Germany . Email: Olaf.Walter@ec.europa.eu; b EaStCHEM School of Chemistry , The University of Edinburgh , Joseph Black Building, David Brewster Road , Edinburgh , EH9 3FJ , UK . Email: Polly.Arnold@ed.ac.uk

## Abstract

Neptunium complexes in the formal oxidation states II, III, and IV supported by cyclopentadienyl ligands are explored, and significant differences between Np and U highlighted as a result. A series of neptunium(iii) cyclopentadienyl (Cp) complexes [Np(Cp)_3_], its bis-acetonitrile adduct [Np(Cp)_3_(NCMe)_2_], and its KCp adduct K[Np(Cp)_4_] and [Np(Cp′)_3_] (Cp′ = C_5_H_4_SiMe_3_) have been made and characterised providing the first single crystal X-ray analyses of Np^III^ Cp complexes. In all NpCp_3_ derivatives there are three Cp rings in η^5^-coordination around the Np^III^ centre; additionally in [Np(Cp)_3_] and K[Np(Cp)_4_] one Cp ring establishes a μ-η^1^-interaction to one C atom of a neighbouring Np(Cp)_3_ unit. The solid state structure of K[Np(Cp)_4_] is unique in containing two different types of metal–Cp coordination geometries in the same crystal. Np^III^(Cp)_4_ units are found exhibiting four units of η^5^-coordinated Cp rings like in the known complex [Np^IV^(Cp)_4_], the structure of which is now reported. A detailed comparison of the structures gives evidence for the change of ionic radii of *ca.* –8 pm associated with change in oxidation state between Np^III^ and Np^IV^. The rich redox chemistry associated with the syntheses is augmented by the reduction of [Np(Cp′)_3_] by KC_8_ in the presence of 2.2.2-cryptand to afford a neptunium(ii) complex that is thermally unstable above –10 °C like the U^II^ and Th^II^ complexes K(2.2.2-cryptand)[Th/U(Cp′)_3_]. Together, these spontaneous and controlled redox reactions of organo-neptunium complexes, along with information from structural characterisation, show the relevance of organometallic Np chemistry to understanding fundamental structure and bonding in the minor actinides.

## Abstract 2

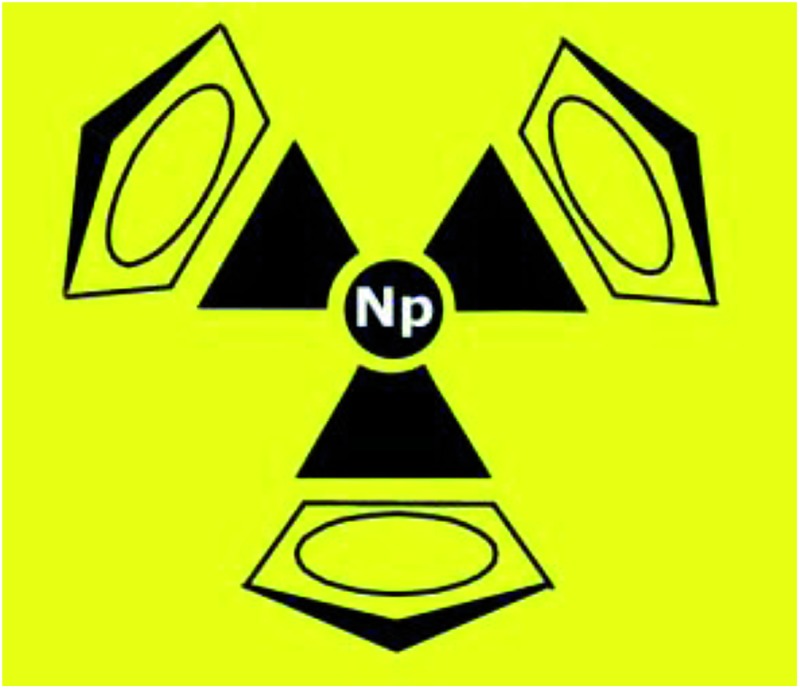
Structural investigation on neptunium cyclopentadienyl organometallic complexes in the formal oxidation states II, III, and IV: similarities and differences between Np and U.

## Introduction

Fifty years have passed since the foundation of organometallic neptunium chemistry in the form of cyclopentadienyl chemistry,^1^ and yet only a handful of complexes have been reported, and even fewer fully characterised.^2–4^ Yet increasingly, combined synthetic/spectroscopic/computational studies are demonstrating how covalently binding, soft, carbocyclic organometallic ligands that form actinide-ligand σ-, π-, δ- and even φ-(back)bonding interactions provide an excellent platform for advancing the fundamental understanding of the differences in orbital contributions and covalency in f-block metal–ligand bonding.^5^ Understanding the subtleties are key to the safe handling and separations of the highly radioactive nuclei.^6–8^ For example, recent quantitative carbon K-edge X-ray absorption spectroscopy (XAS) analyses on the organometallic [An(COT)_2_] (An = Th, U), “actinocenes” provided the first experimental evidence for extensive φ-orbital interactions in thorocene, and remarkably little in the U analogue, with contrasting trends in orbital mixing.^9^ Furthermore, a combination of experimental and QTAIM computational comparisons of [M(L^Ar^)X] (M = Sm, U, Np; X = Cl, I; L^Ar^ = dianionic arene-bridged *trans*-calix[2]benzene[2]pyrrole^10^) showed significant differences (up to 17%) in orbital contributions to M–L bonds between the Ln and An analogues, and that the covalency in the Np–ligand bonding arises from spatial orbital overlap rather than a coincidental energy degeneracy.^4^ The work also demonstrated differences between U and Np in their reaction chemistry, such as the stability of the Np^II^ formal oxidation state or the reduction of Np^IV^ to Np^III^ upon complexation.

Organoneptunium chemistry has relied heavily on the ubiquitous cyclopentadienyl ligand, Cp = (C_5_H_5_)^–^, as a strongly binding, sterically demanding yet flexible, monoanion, and focused almost exclusively on Np^IV^ complexes. The first evidence for the formation of [Np(Cp)_3_X] (X = Cl, F) came from a radiochemical synthesis, *i.e.* the irradiation of a ^238^U complex with thermal neutrons,^1^ and [Np(Cp)_3_X] (X = Cl, F) were subsequently reported from standard chemical routes as thermally robust, volatile complexes.^11,12^ Baumgärtner *et al.* reported the first homoleptic organoneptunium complex, tetrakis(η^5^-cyclopentadienyl)neptunium(iv), [Np(Cp)_4_], from treatment of NpCl_4_ with excess KCp in benzene.^13^ Many of the earliest studies on neptunium cyclopentadienyl complexes also had the aim of exploring covalency in the bonding, using the fact that Np is a Mössbauer active nucleus. However, not all the studies agreed. The first Mössbauer studies on [Np(Cp)_4_] and [Np(COT)_2_] suggested less interaction between the central ion and the Cp ligands but appreciable covalency in the Np–COT bonding.^14^ On the other hand, Bohlander^12^ reported the isomer shift of the Np nucleus in [Np(Cp)_4_] closely approaches that of the record-breaking, covalent [Np(COT)_2_], thus being the most covalent Np–Cp derivative. Finally, from analysis of the isomer shifts Karraker^14,15^ stated that there were smaller covalent bonding contributions in [Np(Cp)_4_] than [Np(Cp)_3_Cl], whereas Adrian^16,17^ concluded the opposite.

The redox properties of the element play a pivotal role in neptunium chemistry as it conventionally exhibits five oxidation states in compounds, from +3 to +7.^18^ Recently, we reported that a formally Np^II^ complex Np(L^Ar^)(dme) supported by L^Ar^, the *trans*-calix[2]benzene[2]pyrrole is accessible; black solutions were sufficiently stable (up to 90 minutes) for spectroscopic analyses but crystals were too small for single crystal diffraction analyses.^4^ Somewhat surprisingly, given the increased stability of the Np^III^ oxidation state compared to U^III^, this work was also the first to report single crystal structural studies on organometallic Np^III^ complexes. To date, the only organometallic Np^IV^ complexes characterised by single crystal X-ray diffraction are neptunocene [Np(C_8_H_8_)_2_],^2,19^ [Np(Cp)Cl_3_(OPPh_2_Me)_2_],^20^ [Np(Cp)_3_(OPh)],^21^ and two examples of the Cp_3_Np-functionalised adduct [(UO_2_)(THF)(H_2_L)] (L = ‘Pacman’ Schiff-base polypyrrolic macrocycle),^3^ in which we studied the Cp_3_Np coordination to one oxo group of the uranyl dication to compare the degree of electron transfer *via* the oxo-bridge between U, Np, and Pu cations. Structurally characterised Np^III^ organometallics are still limited to the [M(L^Ar^)X] complexes we reported.^4^ Modern characterising data including ^1^H NMR spectroscopic data have been reported for just a couple of complexes.

Many routes to solvated and base-free U^III^(Cp)_3_ complexes exist, but only the THF solvate of [Np^III^(Cp)_3_] has been reported to date, and was made from treating [Np(Cp)_3_Cl] with potassium metal and catalytic naphthalene in refluxing THF for a few days. The product was first assigned as the tris THF solvate [Np(Cp)_3_(THF)_3_]^14^ but subsequent IR, FIR and UV-vis-NIR spectroscopic analyses suggested the constitution of an 1 : 1 Lewis base adduct [Np(Cp)_3_(THF)] analogously to that of uranium.^12^ Attempts to desolvate it by heating samples *in vacuo* led to significant decomposition.^14,22^

Herein, we report the synthesis and structural characterisation of a series of Np^III^ cyclopentadienyl complexes, and their reduction chemistry, both spontaneous and directed. Structural changes are discussed in relation to the neptunium formal oxidation state and nature of the ligands, as the majority of the complexes have been structurally characterised by single crystal X-ray diffraction.

## Results

### Syntheses

**Scheme 1:**
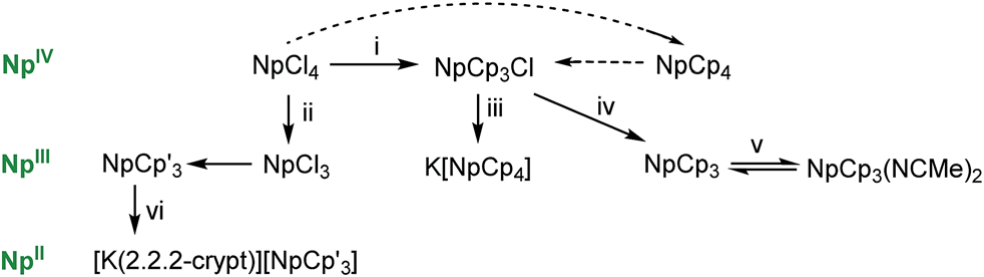
Synthetic routes to neptunium complexes presented in this work. **NpCp_3_** is obtained by reduction from **NpCp_3_Cl** and readily forms the MeCN stabilised solvate. The silylated analogue **NpCp′_3_** (Cp′ = C_5_H_4_SiMe_3_) is better obtained from the reaction of NpCl_3_ with KCp′ and can be reduced to its K salt. Dotted lines indicate literature procedures. Key: (i) excess KCp, PhMe; (ii) Na/Hg, Et_2_O, –NaCl; (iii) KCp, –KCl; (iv) Na/Hg, Et_2_O, –NaCl; (v) excess MeCN; (vi) KC_8_, 2.2.2-cryptand, THF/Et_2_O, –8C.

All the syntheses start from NpCl_4_. This can be directly transformed into [Np(Cp)_4_], **NpCp_4_** by the reaction of NpCl_4_ with an excess of KCp in toluene.^13^ Single crystals of **NpCp_4_** grow in the supernatant during a prolonged extraction of the crude product with pentane. The spectroscopic data agree with the literature reports.^12,23^ **NpCp_4_** itself can be – from a reaction with NH_4_Cl^11,12^ – converted to [Np(Cp)_3_Cl], **NpCp_3_Cl** which is an ideal starting material for the synthesis of [Np(Cp)_3_], **NpCp_3_**. The reduction of **NpCp_3_Cl** by Na/Hg in Et_2_O affords **NpCp_3_** as its diethyl ether solvate [Np(Cp)_3_(Et_2_O)], Scheme 1. This solvent molecule is labile and can be easily removed in vacuum to afford the solvent free **NpCp_3_** which is only sparingly soluble in non-coordinating solvents due to the polymeric nature of the molecular structure (see below) but dissolves slowly in Et_2_O, THF, or MeCN, again forming solvates [Np(Cp)_3_(Et_2_O)], [Np(Cp)_3_(THF)], or [Np(Cp)_3_(NCMe)_2_], **NpCp_3_(NCMe)_2_**, respectively.

Crystals of [Np(Cp)_3_(Et_2_O)] could not be analysed *via* X-ray diffraction as during the crystal mounting procedure they lose coordinated Et_2_O solvent molecule readily. Similarly, no stable adducts of a lanthanide analogue [Ln(Cp)_3_(OEt_2_)] have yet been reported. However, single crystals of the bis-acetonitrile adduct **NpCp_3_(NCMe)_2_** have been grown from acetonitrile solution at RT and studied by X-ray diffraction.

The reaction of **NpCp_3_Cl** with 1.1 equiv. of KCp in THF does not lead to the simple Cl substitution product **NpCp_4_**. After 4 d reflux and evaporation of the solvent, *n*-pentane extraction recovered half of the starting material **NpCp_3_Cl** (49%), and a subsequent extraction with Et_2_O afforded maroon single crystals of the new, reduced, Np^III^ complex K[Np(Cp)_4_], **K[NpCp_4_]** in 37% yield. When the reaction is repeated without heating the mixture, no soluble, molecular product can be isolated.

For the synthesis of [Np(C_5_H_4_SiMe_3_)_3_], **NpCp′_3_** another reaction pathway was followed as no starting material [Np(C_5_H_4_SiMe_3_)_3_Cl] was available: NpCl_3_ is generated *in situ* from the reaction between NpCl_4_ and excess Na(Hg) in diethyl ether at room temperature. The reaction between this NpCl_3_ and three equivalents of K(C_5_H_4_SiMe_3_) in diethyl ether at room temperature afforded the target **NpCp′_3_**. Green, crystalline **NpCp′_3_** is deliquescent under 1 atm of *n*-pentane vapour at room temperature but single crystals can be isolated reproducibly by evaporation and cooling of pentane solutions.

The reduction of a solution of **NpCp′_3_** with KC_8_ was carried out similarly to the method originally described for the synthesis of the thermally unstable [K(2.2.2-cryptand)][U(C_5_H_4_SiMe_3_)_3_] by Evans *et al.*,^24^ but the crystallization temperature maintained lower, at –78 °C. A trial reduction of **NpCp_3_** confirmed that no compound could be isolated even at the coldest achievable reaction temperatures. In THF/Et_2_O the mixture immediately turns very intense dark brown on contact with the solid reducing agent, and small, shiny black crystallites with the assumed composition K(2.2.2-cryptand)[Np(Cp′)_3_] appear in the filtrate after approx. 1 h of storage at –78 °C. Several potentially single crystals of suitable size for an X-ray diffraction study were analysed but only very weak diffraction was observed as the crystals had degraded during the radiologically protective mounting procedure. Due to the high sensitivity of the compound we were not able to determine the structure of the reduced product from this reaction or to measure any spectra.

### Spectroscopy

The ^1^H and ^1^H–^13^C gHMQC NMR spectra of **NpCp_3_** in THF-*d*_8_ show only one resonance for the Cp ring protons at *δ*_H_ = –9.65 ppm and the respective ^13^C shift of *δ*_C_ = 150.4 ppm. The ^1^H NMR spectrum of **NpCp′_3_** in toluene-*d*_8_ solution contains three paramagnetically contact-shifted resonances between –1.38 ppm (the SiMe_3_ protons) and –9.51 ppm in a 9 : 2 : 2 ratio, implying the identical bonding mode of the three ligands. The spectrum acquired in THF-*d*_8_ solution appears similar, the resonances are slightly shifted downfield (*δ*_H_ = –0.6 to –9 ppm) suggesting an interaction with the THF solvent. The ^1^H NMR spectrum of the sparingly soluble **K[NpCp_4_]** in THF-*d*_8_ contains one broad resonance at –11.9 ppm, again showing the identical coordination behaviour of the Cp rings in solution. The solubility of **K[NpCp_4_]** in THF is however too low to observe measurable absorptions in the UV-vis-NIR spectra.

For the Np^III^ complexes, all the Cp ring proton resonances are observed at *ca.* –10 ppm suggesting an electronically similar environment in each complex. Where no correlated spectra are reported here then the complex decomposes in the fluoropolymer NMR-tube liners before the spectra could be recorded.^25^

The ATR spectrum of **NpCp_3_** features several characteristic vibrations of the Cp rings, which correlate well with the previously reported IR data of the complexes [M(Cp)_3_] (M = U, Pu, Am, and Tm).^26^ Indeed, the similarity of the values of the entire series shows the very comparable constitution of the complexes. The most characteristic bands are the set of four absorptions at 666, 611, 581 and 519 cm^–1^.^27^ ATR-FTIR spectra of **K[NpCp_4_]** show very similar Cp ring vibrations to those previously described for **NpCp_3_** with a slight shift to lower energy of the vibrations in **K[NpCp_4_]** *vs.* **NpCp_3_** agreeing with the higher overall negative charge in the complex **K[NpCp_4_]**.

### Molecular structures

**Fig. 1:**
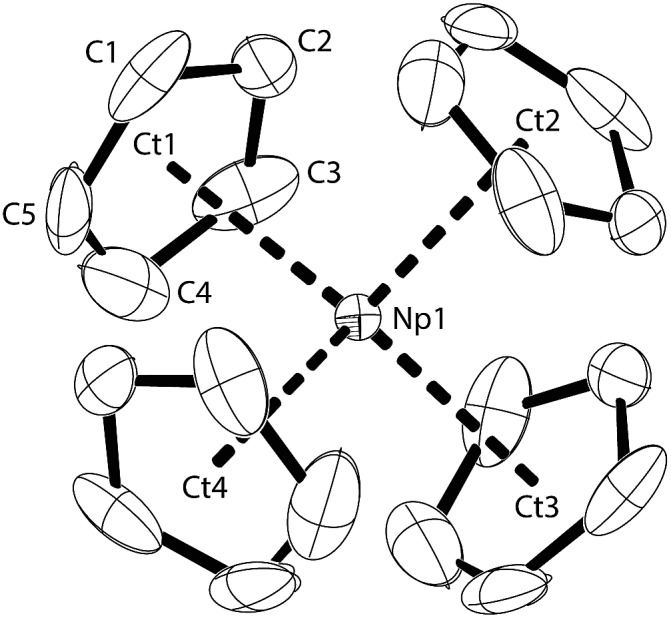
Thermal ellipsoid drawing (50% probability for non-H atoms) of **NpCp_4_** in the solid state. H atoms omitted. Selected bond lengths [Å] and angles [°] for **NpCp_4_**: Np1–Ct 2.551(2), Np1–C 2.78(1) to 2.83(1), Ct–Np1–Ct 109.4(2).

The molecular structure of **NpCp_4_** is here reported for the first time. Dark red single crystals were obtained by extraction with pentane over several days. The compound is kinetically stable. X-ray diffraction analyses revealed an ideal tetrahedral environment of the Np centre, shielded with the four Cp rings in an isostructural complex to its Th^28^ or U^29^ analogue (Fig. 1).

Across the row of the isostructural [An(Cp)_4_] (An: Th, U, Np), in line with the actinide contraction the cell volume decreases from 802 Å^3^ (Th) to 786 Å^3^ (U) to 775 Å^3^ (Np). Furthermore, a shrinking of the entire molecule is expressed by a decreasing An–Cp ring centroid distances. These are found to be 2.606 Å for Th, whereas in the U analogue they are determined to 2.588 Å and for the here presented Np complex they are 2.551 Å, again shorter. The shrinking parallels the decrease of the ionic radii; this implies that the nature of the bonding in the complexes in this row is comparable and even if covalency plays a role it does not affect the bond lengths in the complexes significantly.

Dark-brown, almost black single crystals of **NpCp_3_** suitable for X-ray crystal structure determination were obtained from a 3.5% v/v diethyl ether in *n*-pentane solution stored at room temperature for 7 d.

**Fig. 2:**
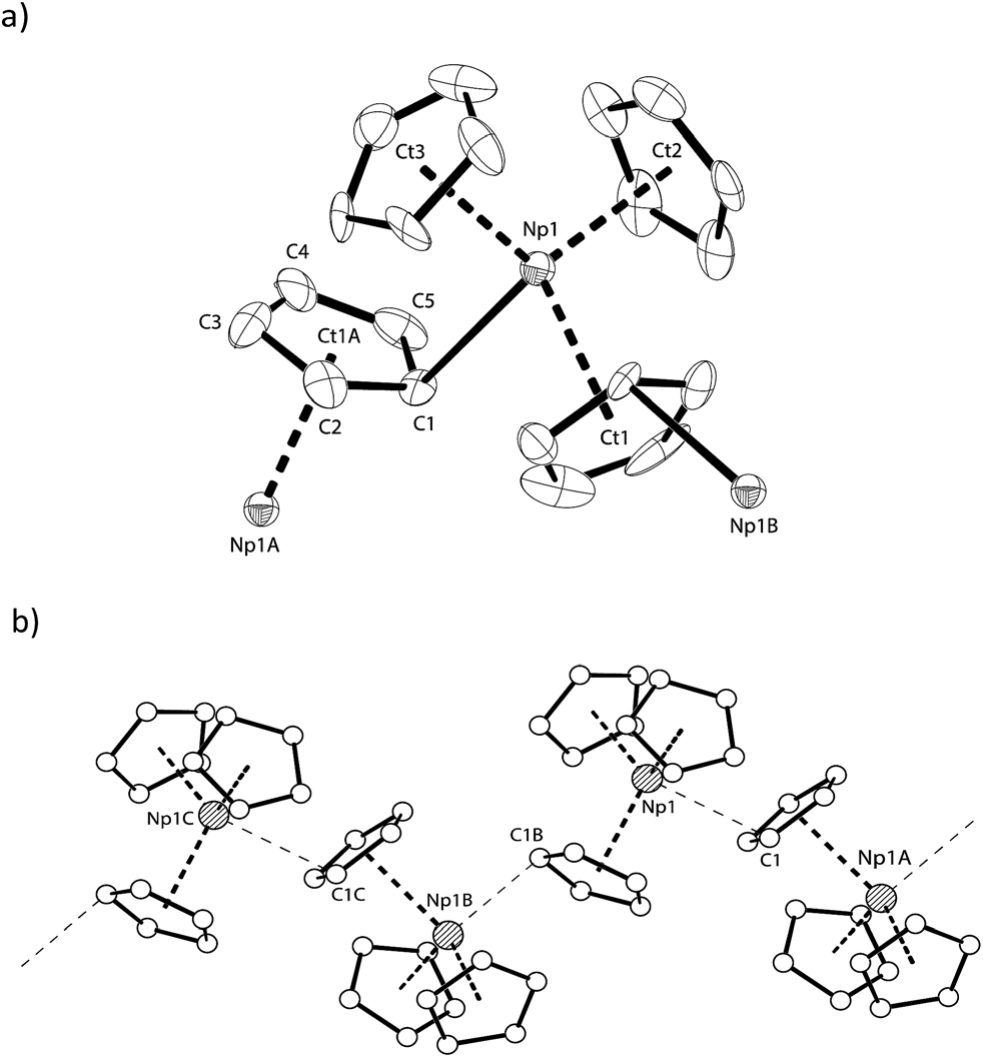
(a) Thermal ellipsoid drawing (50% probability for non-H atoms) of a portion of the polymeric chain formed by [Np(Cp)_3_] in the solid state. H atoms omitted. Selected bond lengths [Å] and angles [°] for **NpCp_3_**: Np1–C1 2.815(11), Np1–Ct1 2.587(5), Np1–Ct2 2.419(6), Np1–Ct3 2.561(10), Ct1–Np1–Ct2 112.23(19), Ct1–Np1–Ct3 113.66(19), Ct2–Np1–C3 115.1(2), Ct1–Np1–C1 94.5(3), Ct2–Np1–C1 102(5), Ct3–Np1–C1 110.7(3), Np1–C1–Np1A 167.5(3), φ(C1–Np1–Ct1∠Ct2–Np1–Ct3) 91.30(18), φ(C1–Np1–Ct3∠Ct1–Np1–Ct2) 82.9(2); (b) representation of the zig–zag chain arrangement in [Np(Cp)_3_]. Symmetry generated atom names are labelled with A, B and C.

The solid-state structure of **NpCp_3_** shows a polymeric structure motif. All the three Cp rings are bound η^5^ towards the central Np^III^ atom. Due to its Lewis acidity coordinative saturation is achieved by additional η^1^-coordination to one of the Cp rings of another **NpCp_3_** unit resulting in one μ-η^5^,η^1^-coordinated bridging cyclopentadienyl group, Fig. 2a, establishing an overall polymeric zig–zag structure with a Np1–C1–Np1A angle close to 170, Fig. 2b. This is in agreement with the structures of isostructural [Ln(Cp)_3_] complexes.^30–33^ In this coordination environment a distorted tetrahedral geometry around the Np^III^ centre is established with a strong Np–C interaction to the C-atom to which the η^1^ coordination is established (Np–C1 2.815(11) Å) whereas the bond distance between the Np1 atom and the carbon atoms C2 (3.266(9) Å) and C5 (3.552(10) Å) may be considered as non-bonding. The geometry around the cation in **NpCp_3_** is more closely aligned with the larger rare earth analogues that have more electron-rich Cp rings, [Ln(C_5_H_4_Me)_3_]_4_ (Ln = La,^34^ Ce,^35^ Nd^36^). These all show μ-η^5^,η^1^-binding for each cyclopentadienyl ligand. However, some of the published data were recorded at room temperature and are less well resolved. In order to discuss fully the differences between the isostructural complexes of the type [M(Cp)_3_] (M: Ln, An) it will be necessary to re-determine the solid state structures of the corresponding Ln complexes.

**Fig. 3:**
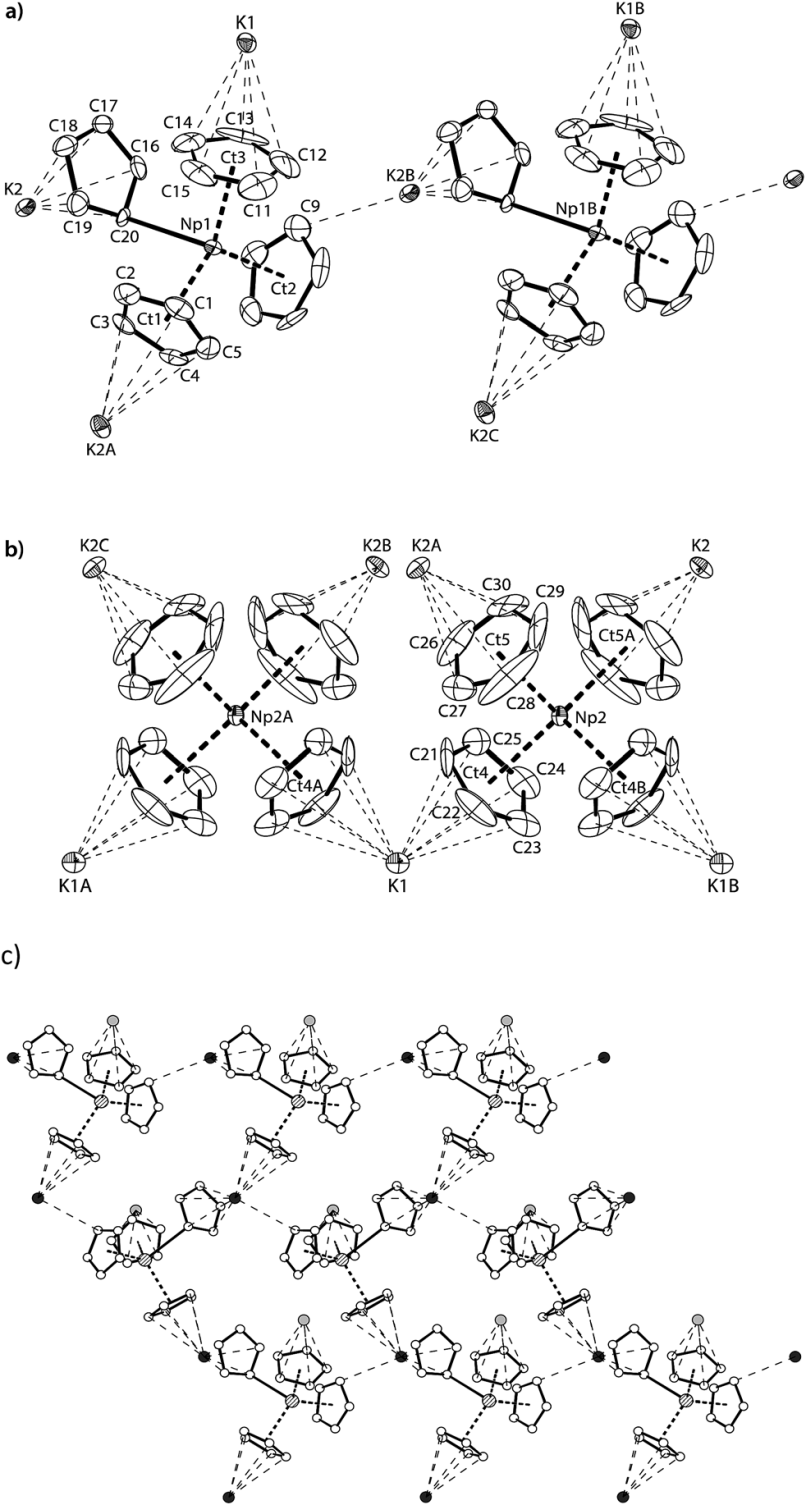
(a) Thermal ellipsoid drawing of a portion of the polymeric structure type K[Np(η^5^-Cp)_3_(η^1^-Cp)] formed by Np^III^ cations A, and (b) the polymeric sheet-like structure type K[Np(η^5^-Cp)_4_] formed by Np^III^ cations B. Thermal ellipsoids for (a) and (b) are at 50% probability. (c) Ball and stick drawing of part of the 2D-polymeric sheet structure formed by the type A Np^III^ cations and K2. Atom colours: C (white), K1 (light grey), K2 (dark grey), Np1 (filled with lines). Hydrogen atoms and lattice diethyl ether molecules are omitted for clarity. Symmetry generated atom names are labelled A, B and C. Selected distances [Å] and angles [°] for **K[NpCp_4_]**; Np1–C20 2.752(7), Np1–Ct1 2.527(4), Np1–Ct2 2.516(4), Np1–Ct3 2.493(6), K1–Ct3 3.067(6), K1–Ct4 3.013(5), K2–C9 3.197(10), K2–C16 3.049(8), K2–C20 2.976(7), K2A–Ct1 3.003(5), K2–Ct5A 2.884(7), Ct1–Np1–Ct2 115.50(13), Ct1–Np1–Ct3 118.92(16), Ct2–Np1–Ct3 117.19(17), Ct1–Np1–C20 96.53(16), Ct2–Np1–C20 99.01(17), Ct3–Np1–C20 103.51(19), Np2–Ct4 2.631(5), Np2–Ct5 2.645(7), Ct4–Np2–Ct5 109.2(2), Ct4–Np2–Ct4B 109.6(2), Ct4–Np2–Ct5A 110.5(2), Ct5–Np2–Ct5A 107.9(3), Ct4–K1–Ct4A 104.67(19), φ(Ct4–Np2–Ct5∠Ct4B–Np2–Ct5A) 88.7(2), φ(Ct4–Np2–Ct4B∠Ct5–Np2–Ct5A) 90.9(3).

Red-brown crystals of **K[NpCp_4_]** suitable for single crystal X-ray diffraction analysis were grown from a diethyl ether solution stored at room temperature for *ca.* 100 h. The asymmetric unit consists of 1.5 molecules of **K[NpCp_4_]** and 0.5 molecule of a heavily disordered diethyl ether molecule residing on the crystallographic *C*_2_ axis. **K[NpCp_4_]** is also polymeric in the solid-state, with all Cp ligands forming bridging interactions of either η^1^ to another Np atom or η^5^ to a K or Np cation. This results in two different types of Np coordination geometry arising from the bridging modes, Fig. 3. The coordination environment around the first Np^III^ cation, labelled A, is (η^5^-Cp)_3_(η^1^-Cp), which closely mirrors that of the parent complex **NpCp_3_**; that around the second Np^III^ cation, labelled B, is (η^5^-Cp)_4_ comparable to the coordination observed for the four Cp rings in **NpCp_4_**. To our knowledge, this is the first instance of a Cp complex showing two different types of metal coordination geometries in the same crystal. This behaviour might be explained by the high coordinative flexibility of the relatively large An cations. Very few f-block complexes have comparable solid state structures. The complex [Ce(C_5_H_4_Me)_3_]_4_ forms a tetramer in the solid state with (η^5^,η^1^-Cp) anions bridging,^35^ and one uranium complex was published very recently; [K(2.2.2-cryptand)][U(Cp′)_4_],^37^ also shows an (η^5^-Cp)_3_(η^1^-Cp) geometry. The An^III^(η^1^-Cp) M–C distance is 2.752(7) Å for Np and 2.776(2) for U, consistent with the ionic radius difference (six-coordinate Np^3+^ = 1.01 Å; U^3+^ = 1.025 Å).^38^

The Np(A) and (B) type cations both form polymeric chain structures, which connect to each other, and into a 3D network *via* further K cation interactions. For Np(A), Fig. 3a, there are three distinct Cp binding modes for the four Cp ligands: one K(η^5^-)Np(η^1^-); one K(η^1^-)Np(η^5^-) (for Ct2); and two K(η^5^-)Np(η^5^-) (for Ct1 and Ct3). The second type of Np, type B, Fig. 3b, displays η^5^ binding of all the Cp ligands arranged in an irregular tetrahedral fashion around the Np^III^ centre. The average separation between the Np atoms and the η^5^-Cp ring centroids in these molecules is slightly longer in accordance with the greater steric encumbrance at the Np centre (2.635(1) in B *vs.* 2.507(5) Å in A-type). Accordingly, the C-atoms for the fourfold η^5^-coordinated Np centres show larger Np–C distances between 2.835(12) to 2.955(9) Å. The range of Np–C distances to like in **NpCp_3_** coordinated Np centre Np(A) for the η^5^-bound carbon atoms is with 2.732(13) to 2.842(9) Å comparable.

There are also two different types of potassium coordination. Cation K1 shows only η^5^-Cp binding, with long K–Cp (centroid) distances of 3.013(5) and 3.067(6) Å. Cation K2 shows both η^1^-Cp binding (to C9 with a distance of 3.197(10) Å) and much closer η^5^-Cp binding than K1, with K–Cp (centroid) distances of 2.884(7) and 3.003(5) Å. These latter are more typical K–Cp distances. There are also molecules of diethyl ether present in the lattice, but no close contacts to metal centres are evident, Fig. 3c.

Np(A) in **K[NpCp_4_]** exhibit very similar coordination behaviour to **NpCp_3_**. The zig–zag chains formed by the A-type Np^III^ units are only slightly more bent in K[Np(Cp)_4_] (Np–C–Np 149.8(3)°) than in **NpCp_3_** (156.9(5)°). The mean distance between the Np(A) atoms and the centre of the η^5^-coordinated Cp rings is in average 2.51(1) Å and absolutely comparable to the one in **NpCp_3_** with 2.52(1) Å. A bigger difference is observed for the bond length C–Np of η^1^-coordinated C-atoms: in **NpCp_3_** at 2.815(11) Å it is significantly larger than for Np(A) in **K[NpCp_4_]** with 2.752(7) Å. The different coordination behaviour of the Cp rings (binding η^1^ to the Np atoms but η^5^-coordination to a K cation in **K[NpCp_4_]** and to a Np in **NpCp_3_**) is attributed to poorer competition by K for coordination than Np, allowing the Cp ring to enable a stronger interaction towards the metal on the opposite site.

Np(B) in **K[NpCp_4_]** with its four η^5^-coordinated Cp rings placed in an identical coordination environment to **NpCp_4_** with the difference of the charge on central Np atoms. According to the higher charge the mean centre of Cp ring to Np distance in **NpCp_4_** is with 2.551(1) Å about 0.08 Å shorter than for Np(B) in **K[NpCp_4_]** where it is determined to 2.635(1) Å. This effect can be attributed mainly to the change of the ionic radii from Np^III^ to Np^IV^ in an otherwise identical coordination environment; in this case four Cp ligands.

**Fig. 4:**
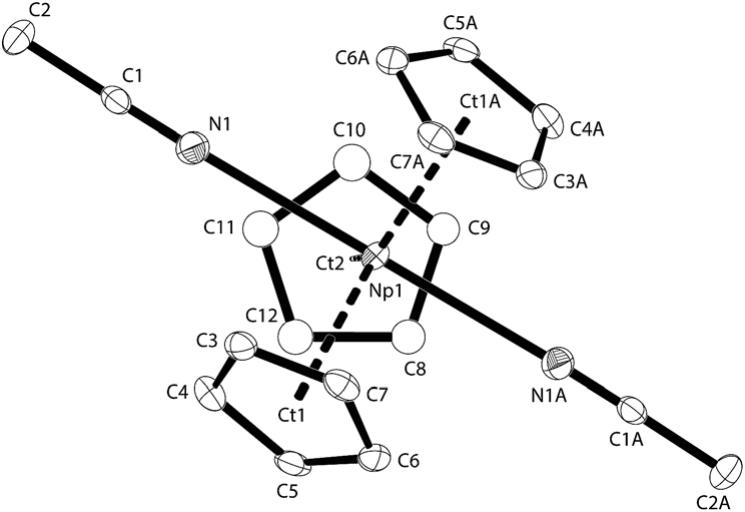
Thermal ellipsoid drawing (50% probability for non-H atoms) of **NpCp_3_(NCMe)_2_** in the solid state. H atoms omitted. Selected bond lengths [Å] and angles [°] for **NpCp_3_(NCMe)_2_**: Np1–N1 2.665(4), Np1–Ct1(a) 2.539(4), Np1–Ct2 2.540(4), N1–C1 1.135(5), C(1)–C(2) 1.465(5), N1–Np1–N1A 178.4(2), Ct–Np1–Ct: 116.9(2), 119.9(2), 123.5(2).

Single crystals of **NpCp_3_(NCMe)_2_** grow from a MeCN solution of **NpCp_3_** as a solution is concentrated under reduced pressure. They are stable enough that they can be transferred on the goniometer and the single crystal solid state structure measured (Fig. 4).

The geometry around the Np atom in the molecular structure of **NpCp_3_(NCMe)_2_** can be generally described as distorted trigonal bipyramidal with three Cp in η^5^-coordination mode exhibiting the trigonal plane around the Np atom whereas the two MeCN ligands are forming occupying its apical positions. In the molecule there is a two-fold axis passing through the Np atom. This results in the nearby linear arrangement of the two acetonitrile ligands exhibiting a Np angle of 178.4(2)°. The low steric demand of the MeCN ligands enables a close to ideal arrangement of the three Cp rings in one plane around the Np centre showing a sum of angle of 360.3° (Ct–Np1–Ct) with a deviation out of the plane consisting of the three centres of the Cp rings plus the Np atom of less than 0.005 Å. Accordingly, the bond distances between the Np atom and the centres of the Cp rings are identical with 2.539(2) and 2.540(2) Å. These findings compare well to the metrics for the series of LnCp_3_(nitrile)_2_ complexes that have been structurally characterised.^39–43^ For the actinides however, only some cationic U^IV^ complexes have been structurally characterised.^44–47^ In these U^IV^ cationic complexes of the type UCp_3_(NCR)_2_^+^ shorter bond distances are found than here for **NpCp_3_(NCMe)_2_** (U–N distances <2.6 Å).

**Fig. 5:**
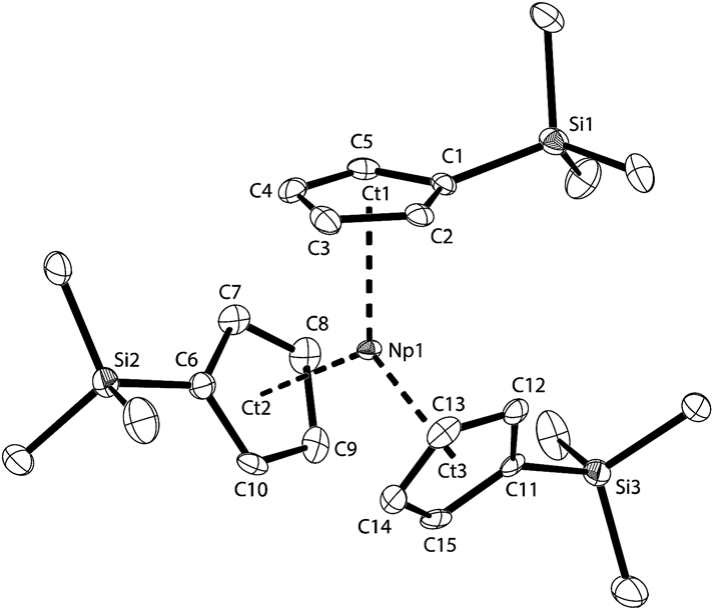
Thermal ellipsoid drawing (50% probability for non-H atoms) of **NpCp′_3_** in the solid state. H atoms omitted. Selected bond lengths [Å] and angles [°]: Np1–Ct1 2.485(2), Np1–Ct2 2.481(2), Np1–Ct3 2.479(2), Np1–C_ar_ 2.734(6) to 2.786(4), Si1–(C1–C5) plane –0.382(8), Si2–(C6–C10) plane –0.109(8), Si3–(C11–C15) plane –0.169(8), Ct1–Np1–Ct2 119.86(8), Ct1–Np1–Ct3 120.46(7), Ct2–Np1–Ct3 119.06(8).

All of the Np^III^ centres described above are coordinatively unsaturated in the absence of ligand bridging, but this situation can be readily changed by the use of sterically more demanding Cp ligands like C_5_H_4_SiMe_3_(Cp′). Olive-green single crystals of **NpCp′_3_** suitable for X-ray diffraction analysis were obtained from cooling a concentrated *n*-pentane solution to –20 °C. The asymmetric unit consists of a single molecule of **NpCp′_3_**. The molecular structure of **NpCp′_3_** is shown in Fig. 5 and consists of the mononuclear Np^III^ complex containing three η^5^ bound Cp′ ligands, with all the Ct(η^5^-Cp′)–Np–Ct(η^5^-Cp′) angles close to 120° and the average Np–Ct(η^5^-Cp′) distance of 2.482(3) Å. This means that in **NpCp′_3_** the Cp rings with the bulky substituents are closer to the metal than in the previously described complexes **NpCp_3_(NCMe)_2_**, **K[NpCp_4_]**, and **NpCp_3_** for which Np–Ct distances of 2.51 Å or 2.54 Å are found. This means that due to the trigonal planar arrangement of the Cp′ ligands with the resulting larger bond angles around the Np^III^ centre in **NpCp′_3_** the metal is able to establish stronger interactions with the more electron rich Cp′ ligands.

The Np1 atom in **NpCp′_3_** lies in plane of the Cp ring centroids with only a minor out-of-plane distortion (0.113(1) Å), affording a nearly trigonal planar (*D*_3h_) geometry which is isostructural with the previously reported uranium complex [U(Cp′)_3_].^48^ The average An–C distance of 2.78(4) Å (2.76(3) Å for U) and An–Ct(η^5^-Cp′) of 2.482(3) Å (2.51(3) Å for U) are the same within standard uncertainties. However, the similarity of the values can be taken as a sign that the arrangement of the three Cp′ ligands is dominated more by steric factors than by ionic radii. The ligands take a trigonal planar coordination around the Np centre, which is not only in agreement with the structure of its U analogue, but as well with the solid state structure found for the small lanthanide cation in [Yb(Cp)_3_]^49,50^ or U(Cp^R^)_3_^–^ containing complexes (where R represents a bulky hydrocarbyl group).^24,42,48,51–60^

## Discussion

### Redox reactivity

In Scheme 1 there are mainly represented two reaction pathways: nucleophilic substitution at the metal centre or reduction. Np^IV^Cp_4_ was prepared readily from the reaction of Np^IV^Cl_4_ with excess of KCp *via* SN reaction. From this a Cp ligand can be abstracted by protonation and so that even Cl^–^ is able to coordinate to the metal forming Np^IV^Cp_3_Cl.

The homoleptic complexes **NpCp_3_** and **NpCp′_3_** are accessible only by reduction: **NpCp_3_** is best produced by reduction of **NpCp_3_Cl** whereas **NpCp′_3_** is well prepared by the *in situ* formation of NpCl_3_ from NpCl_4_ followed by SN reaction at the metal centre leading to **NpCp′_3_**. As the silyl-substituted C_5_H_4_SiMe_3_ anion more effectively stabilises lower oxidation state metal cations the synthesis of the first cyclopentadienyl Np(ii) complex succeeded by reduction of **NpCp′_3_** with KC_8_ in parallel with the results of Evans *et al.*,^24^ the crystallization temperature was lowered to –78 °C but the small, shiny black crystallites appearing in the filtrate after 1 h of storage at –78 °C showed insufficient diffraction properties.

Although the neutral complex [Np(Cp)_4_] has been reported several times to be formed in the reaction between NpCl_4_ and excess KCp in THF,^12^ benzene,^12,13^ or toluene^61^ solution, the reaction reported here between [Np(Cp)_3_Cl] and KCp affords the Np^III^ ate product K[Np(Cp)_4_] giving evidence that in this case Cp^–^ acts in two roles: as reducing agent plus as stabilising ligand for the coordinatively unsaturated Np^III^ ion, dependent on the reaction conditions.

These observations could provide an explanation for the disagreements in the Mössbauer studies on covalency. Adrian observed that Mössbauer spectra of the [Np(Cp)_4_] targets provided by Bohlander contained two low intensity bands arising from the unidentified impurities, which may provide an argument for this study.^16^ The utility of the Cp anion as a reductant is well documented in preparative inorganic chemistry, and an additional equivalent(s) of either NaCp or [MgBr(Cp)] can be conveniently employed to reduce *in situ* the higher oxidation state transition metal and lanthanide precursors and produce metallocenes of the M^II^ centres *i.e.* Cr,^62,63^ V,^63,64^ Ru,^65^ Os,^66^ or Eu.^67^ In actinide chemistry this reactivity is rarer, and the only reported synthesis to date is of the homoleptic complex [^239^Pu(Cp)_3_] from treatment of [Cs_2_(^239^PuCl_6_)] with excess Mg(Cp)_2_.^68^ However, it is pertinent to note that the salt metathesis reactions between [Np(Cp)_3_Cl], and group 1 alkyl- or aryl-anions formed only low yields of [Np(Cp)_3_(*n*-Bu)] and [Np(Cp)_3_Ph] (40–60%) alongside undefined Np^III^ by-products, presumably due to the homolysis of the Np^IV^-alkyl bond.^12,61^

The reported formal potentials summarized in Table 1 show the Np^IV^/Np^III^ couple is intermediate in value between U and Pu in the triad, as would be expected. Cyclic voltammetry experiments have demonstrated that [An(Cp)_3_Cl] (An = U, Np) complexes show reversible one-electron reduction processes at *E*_1/2_ = –1.80 V (U^IV^/U^III^) and –1.29 V (Np^IV^/Np^III^) in THF (*vs.* Fc^+^/Fc).^70^ Early actinide elements (An = Th–U) demonstrate a clearer thermodynamic preference for the +4 over +3 oxidation state and in its organometallic chemistry,^11^ for Np^IV/III^ the preference is more finely balanced. The electrochemical properties of actinide centres in organoactinides are usually considerably affected by ligand environments.^71^

**Table 1:** Formal potentials (V, *vs.* SHE) of An^IV^/An^III^ couples in aqueous solutions^69^

Couple	Formal potential, *E*°′, in V *vs.* SHE		
	1 M HClO_4_	pH 8	1 M NaOH
U^IV^/U^III^	–0.631	–1.95 ± 0.17	–2.78 ± 0.35
Np^IV^/Np^III^	0.155 ± 0.010	–1.13 ± 0.14	–1.88 ± 0.24
Pu^IV^/Pu^III^	0.9821 ± 0.0005	–0.39 ± 0.15	–1.04 ± 0.24

The disproportionation of U^III^ into 0.75 eq. of An^IV^ and 0.25 eq. of An^0^ is well-known, and has been reported for Np^III^.^11,72^ We used a variety of techniques to confirm the formal U^III^ oxidation state in the inverse sandwich complexes [{X_2_U}_2_(μ-η^6^,η^6^-C_6_R_6_)], (in which the arene carries a dianionic charge) (X = bulky aryloxide or amido monoanion, C_6_R_6_ = benzene, toluene, naphthalene, and silylated or borylated arene derivatives) that were formed from the disproportionation of U^III^X_3_ molecules into U^IV^X_4_ and the formal intermediate U^II^X_2_.^72^ More recently, Meyer used computational analyses to confirm the formal U^II^ oxidation state in the arene-supported tris(aryloxide) ate complex [K(2.2.2-crypt)][((^Ad,Me^ArO)_3_mes)U].^73^ Following the report of the +2 oxidation state for uranium in a molecular complex [K(2.2.2-cryptand)][U(Cp′)_3_] by Evans *et al.*,^24,37^ and our report of the relatively stable, formally Np^II^ complex Np(L^Ar^)(dme),^4^ which survives up to 90 minutes in solution and as small near-black crystals, the synthesis of the neptunium homologue **K[NpCp′_3_]** of the U ‘ate-’ complex seemed a reasonable target. While a convenient low-temperature route with radiological protection was devised to afford solutions and crystals of a Np(ii) complex it was insufficiently thermally stable to enable characterisation of the solutions or X-ray data collection on single crystals.

This situation should be even easier moving from Np to Pu which already shows a much more stable M^III^ oxidation state in its complexes.

### Solid state structures

All the complexes presented here, three Np^III^ and one Np^IV^Cp_4_, contain at least three Cp ligands in the coordination sphere of the Np, so that a structural comparison can be performed. In the structures of the Np^III^ complexes **NpCp_3_(NCMe)_2_**, **K[NpCp_4_]**, **NpCp_3_** the Np centres are surrounded by three Cp rings in η^5^-coordination mode. In all these complexes the centre of the Cp ring is placed between 2.51 Å and 2.54 Å distant from the Np atom. However, in **K[NpCp_4_]** there is a second coordination mode of the Np^III^ atoms: besides the coordination known from the **NpCp_3_** (and from the complexes LnCp_3_) consisting of the three already mentioned η^5^-coordinated Cp rings plus one bridging Cp ring establishing an additional μ-η-coordination in **K[NpCp_4_]** there is a NpCp_4_ unit with the Np atom surrounded by four Cp rings all in η^5^-coordination. This situation is comparable to the coordination found in complexes [An^IV^Cp_4_], where in the row from Th over U to Np M to centre of ring distances are found of 2.606 (Th), 2.588 (U), and 2.551 Å (Np), respectively. These values compare to the one of 2.635 Å for the four times η^5^-coordinated Np centres in **K[NpCp_4_]**. Thus one can consider the difference in the ionic radii between Np^III^ and Np^IV^ in an equivalent coordination environment built by in this case four η^5^-coordinated Cp rings to be equal to (2.635 – 2.551=) 0.08 Å.

We note that the CN stretch in the IR spectrum of **NpCp_3_(NCMe)_2_** is observed at 2262 cm^–1^, lower than in the corresponding U cationic complexes which have a stronger M–N interaction.

The trigonal planar arrangement of the three Cp′ ligands [Np(Cp′)_3_] around the Np^III^ centre, analogous to the corresponding U complex raises the possibility that this complex should be able to show comparable redox chemistry to that of U and Th, where the geometry provides suitable orbitals for an additional valence electron to reside. Therefore, it was used as the starting material for the organometallic Np(ii) complex for reduction with KC_8_.

## Conclusions

As could be anticipated, the synthetic chemistry of cyclopentadienyl-supported Np^III^ and Np^IV^ complexes is comparable to that of uranium, with the differences mainly being caused by the less negative reduction potential of the Np^4+^ ion. For the first time a solution-based method for the quantitative formation of green, poorly soluble, but high-surface area, and therefore reactive NpCl_3_ has been demonstrated from reduction of NpCl_4_, and shown to be synthetically useful in anaerobic reactions, even in the absence of strongly coordinating solvents. Complexes **NpCp_3_** and **NpCp′_3_** were synthesized reproducibly in high yields *via* salt metathesis routes from this or from more traditional reduction of the known complex **NpCp_3_Cl**.

One notable example of the greater stability of the Np^III^ ion with respect to U^III^ in these complexes is the overlooked reactivity of **NpCp_3_Cl** with excess KCp, which results in the isolation of the first actinide(iii) tetrakis-cyclopentadienyl complex, **K[NpCp_4_]** under the synthetic conditions previously assumed to afford only the neutral complex **NpCp_4_**. Remarkably, the solid-state structure of **K[NpCp_4_]** exhibits intra-crystal dimorphism; two different types of NpCp_4_ coordination geometries, half of the Np^III^ cations are (η^1^-Cp)(η^5^-Cp)_3_ and half are (η^5^-Cp)_4_, with the two different types of Np^III^ forming separate polymeric chains that are bridged by potassium counter-cations to form the extended polymeric structure. Unexpectedly, this structure may answer the concerns expressed by Adrian *et al.* who reported two similar, but unidentified impurities in samples of **NpCp_4_** that they studied by Mössbauer spectroscopy. Comparison of the structures of **K[NpCp_4_]** and **NpCp_4_** enables a differentiation of the ionic radii of Np^III^ and Np^IV^ in this organometallic environment of 0.08 Å. Complex **NpCp′_3_** shows an even closer contact around the Np atom establishing a trigonal planar coordination environment which is again 0.03 Å smaller but offering further redox chemistry opportunities.

Indeed, potassium reduction at low temperatures of **NpCp′_3_** leads to the formation of very dark-brown crystals of a complex assigned as [K(2.2.2-cryptand)][Np(Cp′)_3_] **K[NpCp′_3_]**; these can be isolated but are less thermally stable than the formally Np(ii) complex [Np(L^Ar^)(dme)] previously reported by us;^4^ single crystals of the putative Np^II^ complex **K[NpCp′_3_]** do not survive for long enough to be encapsulated for radiological protection prior to the collection of diffraction data.

The results presented show that neptunium cyclopentadienyl chemistry can show significant deviations from its uranium congeners, in sharp contrast to previous assertions, and the resulting spectroscopic, redox, and structural investigations provide a significant and deeper understanding of minor actinide chemistry.
