# Supplementary material for: Reduction chemistry of neptunium cyclopentadienide complexes: from structure to understanding
Source: Chem Sci. 2017 Jan 30;8(4):2553–61. doi: 10.1039/c7sc00034k (PMC5431675; doi:10.1039/c7sc00034k)
Supplement: Supplementary file 1 [file SC-008-C7SC00034K-s001.pdf]

Electronic Supplementary Material (ESI) for Chemical Science.
This journal is © The Royal Society of Chemistry 2017

SI

Reduction Chemistry of Neptunium cyclopentadienide complexes: from structure to understanding

Michał S. Dutkiewicz,[a,b] Christos Apostolidis,[a] Olaf Walter,[a]* Polly L. Arnold[b]*

Experimental details

Caution! Compounds containing the ^{237}Np isotope represent a potential health risk owing to α emission ($Q\alpha$ = 4.958 MeV, $t_{1/2}$ = 2.14 × 10^6 years). ^{237}Np decays to ^{233}Pa ($t_{1/2}$ = 26.97 days, *a* = 21 kCi g^{-1}), a β emitter (Q^- = 0.570 MeV) which can result in radiation exposure due to penetration of the gloves and skin. Handling Np isotopes are only to be undertaken in a properly regulated and controlled radiological facility.

General notes

The manipulations with Np radionuclides were conducted in the radiochemical laboratories at the Joint Research Centre (JRC) – Karlsruhe, Germany. Unsealed transuranium compounds were manipulated in dinitrogen filled (99+%), negative-pressure radiological gloveboxes. The glovebox for preparative chemistry was fitted with an automated dual vacuum/argon manifold and standard Schlenk techniques were used. Extraction processes refer to continuous sinter-glass extraction according to the modified literature method for extremely reactive solids.[1] A UV-Vis-nIR optical chamber and an ATR-IR spectrometer were contained within radiological gloveboxes to enable direct measurements. ATR-IR spectra were obtained using Bruker ALPHA FT-IR spectrometer fitted with a single reflection Platinium-ATR (diamond) module. Electronic absorption spectra were acquired in the range of 250-1650 nm at 120 nm × min^{-1} scan rate using Perkin-Elmer Lambda 19 UV/Vis/NIR spectrometer and semi-micro quartz cells (Suprasil®) with a path-length of 10.00 mm. The NMR spectra of the Np compounds were recorded on the Bruker Ascent™ 400 MHz WB NMR/DNP spectrometer equipped with a Bruker Triple Resonance Broad Band Probe (TBI). Degassed fluoropolymer NMR tube liners (4 mm nominal O.D.; 140°C, 6 × 10^{-4} mbar, 12 h) were charged with the liquid samples ensuring that the outer surface remained free from contamination, and sealed. The sealed liner was then transferred into a standard borosilicate glass NMR tube placed in a PVC bag, which was sealed by welding. Chemical shifts were calibrated against residual protio solvent signal and are reported relative to tetramethylsilane (δ = 0 ppm).

Chemicals

Commercially available reagents and solvents were obtained from Sigma-Aldrich Co., ACROS Organics, STREM Chemicals Inc. or ABCR GmbH & Co. KG and used as received unless otherwise stated. Oxygen 6.0N and argon 6.0 N were supplied by basi Schöberl GmbH & Co. KG and the latter purified with Agilent Technologies Big Moisture and Oxygen Traps to achieve sub-5 and sub-1 ppb levels of the corresponding impurities respectively. The anhydrous grade solvents were purified immediately before use by distillation from NaK alloy (K_2Na, 77.28% K) under argon. Deuterated tetrahydrofuran (99.5 atom % D) was stirred with NaK alloy until a persistent pale blue colour of the solvated electrons remains, and vacuum-transferred prior to use. Commercial 2.2.2-cryptand (4,7,13,16,21,24-hexaoxa-1,10-diazabicyclo-[8.8.8]-hexacosane) was dried and recrystallized from *n*-hexane prior usage. Anhydrous $NpCl_4$,[2, 3] KCp,[4] [K(CpTMS)] ([K(Cp')]),[5] [Np(Cp)$_4$],[6] and [Np(Cp)$_3$Cl] [7] were prepared according to the literature.

Compounds

[Np(Cp)$_3$]; NpCp$_3$

A 25-mL Schlenk tube was charged with [Np(Cp)$_3$Cl] (230.6 mg, 0.493 mmol), solid sodium mercury amalgam (248 mg, 5.03%, 1.1 eqv.) and mercury metal (2.246 g). Diethyl ether (10 mL) was added and the reaction mixture was stirred vigorously for 12 h. During this time a dark green/grey suspension formed and the dichroic, dark brown to olive green supernatant isolated by filtration (fritted glass disk, 10-16 µm porosity). The filter cake containing the ether-solvate [Np(Cp)$_3$(OEt$_2$)] **NpCp$_3$E**, was extracted with diethyl ether until no visible change in fresh eluent colour and the combined extracts evaporated to dryness under vacuum (i). The resulting black solid was extracted with 3.5% $_{v/v}$ diethyl ether in *n*-pentane for 160 h (ii). Dark brown, almost black single crystals of unsolvated [Np(Cp)$_3$], **NpCp$_3$** were collected and dried briefly under vacuum (20 °C, <5 × 10^{-3} mbar); isol. yield 160-168 mg, 75-79 %. MF = $C_{15}H_{15}Np$ MW = 432.33(2) g mol^{-1}.

^{1}H NMR (THF-*d*$_8$, 291.8 K, 400.34 MHz): δ_H = -9.65 (s, 15H) ppm. ^{1}H-^{13}C gHMQC (THF-*d*$_8$,293.4 K, 400.34 MHz): {δ_H, δ_C} = {-9.65, 150.4} ppm. ATR-IR: ν = 3084 w (ν_{CH}), 3068 w, 2957 vw, 2957 vw, 2924 vw, 2852 vw, 1436 (ν_{CC}), 1350 w, 1307 w, 1238 m, 1184 w, 1125 w, 1065 w (δ_{CC}), 1004 m/s ($\delta_{CH||}$), 878 w, 839 w, 757 s ($\delta_{CH\perp}$), <u>666 m (sh)</u>, 617 m, <u>611 m</u>, <u>580 m</u>, <u>519 m</u> cm^{-1}. Underlined stretches attributed to characteristic fingerprint manifold for complexes containing {Np^{III}(Cp)$_3$} unit. Radiological concerns prohibited combustion-based evaluation of the C and H content of **NpCp$_3$**.

[Np(Cp')$_3$]; NpCp'$_3$

A suspension of NpCl$_3$ is first prepared *in situ*: A 25-mL Schlenk tube was charged with NpCl$_4$ (109.9 mg, 0.290 mmol), solid sodium mercury amalgam (139 mg, 5.03%, 1.05 equiv.) and mercury metal (1.262 g). Diethyl ether (8 mL) was added and the reaction mixture was stirred vigorously for 24 h. During this time the red solid was consumed and an insoluble greenish precipitate formed. To this suspension was added solid [K(Cp')] (153.4 mg, 0.870 mmol) at room temperature, leading to an immediate colour change of the reaction mixture to dark green. The reaction mixture was allowed to stir for further 4 h and the solvent stripped under reduced pressure. The resulting residue was extracted with *n*-pentane (12 mL) and the dark green supernatant isolated by syringe filtration (silanized glass fibre membrane, 1.0 µm porosity). Flash evaporation of the solvent under vacuum afforded [Np(Cp')$_3$], **NpCp'$_3$**, in the form of olive green crystalline solid. The crystals were collected and dried shortly under vacuum (20 °C, <5 × 10^{-3} mbar); isol. yield 177 mg, 94 %. MF = $C_{24}H_{39}NpSi_3$ MW = 648.87(3) g mol^{-1}.

^{1}H NMR (THF-*d*$_8$,293.2 K, 400.34 MHz): δ_H = -0.62 (s, 27H, Si(C<u>*H*$_3$</u>)$_3$), -8.81 (s, 6H, C<u>*H*</u>), -8.98 (s, 6H, C<u>*H*</u>) ppm. ^{1}H-^{13}C gHMQC (THF-*d*$_8$,293.5 K, 400.34 MHz): {δ_H, δ_C} = {-0.62, -6.1}, {-8.73, 154.6}, {-8.93, 158.6} ppm. ^{1}H NMR (toluene-*d*$_8$, 292.8 K, 400.34 MHz): δ_H = -1.38 (s, 27H, Si(C<u>*H*$_3$</u>)$_3$), -8.60 (s, 6H, C<u>*H*</u>), -9.51 (s, 6H, C<u>*H*</u>) ppm. ^{1}H-^{13}C gHMQC (toluene-*d*$_8$, 293.1 K, 400.34 MHz): {δ_H, δ_C} = {-1.38, -14.1}, {-8.61, 165.1}, {-9.47, 166.9} ppm. Radiological concerns prohibited combustion-based evaluation of the C and H content of **NpCp'$_3$**.

[Np(Cp)$_3$(NCMe)$_2$]; NpCp$_3$(NCMe)$_2$

A 10-mL glass ampoule fitted with a greaseless valve was charged with dark-brown, solid **NpCp$_3$** (16.1 mg, 37.2 µmol) and acetonitrile (3.5 mL). The ampoule was shaken (orbital shaker, 2800 rpm) for *ca*. 12 min, affording complete consumption of the dark-brown material and forming a red suspension in a pale green solution. The solution was isolated by syringe filtration (PTFE membrane, 0.45 µm) and concentrated under reduce pressure to *ca.* 1 mL with cooling to (0 °C) to afford pale green crystals of the target product [Np(Cp)$_3$(NCMe)$_2$], **NpCp$_3$(NCMe)$_2$** which were suitable for single crystal X-ray diffraction analysis. However, the yield could not be determined as a flocculant red powdery by-product also precipitates at the same time, and the two could not be separated.

Reduction reactions

K[Np(Cp)$_4$]; **KNpCp$_4$**

A 25-mL Schlenk flask was charged with [Np(Cp)$_3$Cl] (181.5 mg, 0.420 mmol) and potassium cyclopentadienide (48.1 mg, 0.462 mmol). THF (ca. 20 mL) was added and the reaction mixture was stirred vigorously for 1 h at room temperature. During this time no visible precipitate was produced and the reaction mixture was further heated under gentle reflux for a period of 96 h. A reddish brown precipitate formed and the colour of the supernatant changed to dark maroon. The suspension was evaporated to dryness under vacuum (25 °C, <5 × 10^{-3} mbar, 3 h) and the resulting dark brown solid extracted with *n*-pentane (36 h) and subsequently with diethyl ether (*ca.* 100 h). The *n*-pentane extraction separated essentially pure [Np(Cp)$_3$Cl] (88.4 mg, 48.7 %), whilst the following one afforded K[Np(Cp)$_4$], **KNpCp$_4$,** as maroon crystals suitable for single crystal X-ray diffractometry; isol. yield (94.6 mg, 36.9 %). MF = $C_{20}H_{20}$KNp (C_4H_{10}O) MW = 610.64(3) g mol^{-1}.

^{1}H NMR (THF-d_8 ,293.2 K, 400.34 MHz): δ_H = -11.95 (s, 20H) ppm. ATR-IR: ν = 3084 w (ν_{CH}), 3059 w (sh), 2967 vw, 2925 vw, 2853 vw, 1438 (ν_{CC}), 1358 w, 1308 w, 1238 m, 1182 w, 1124 w, 1066 w (δ_{CC}), 1009 m/s ($\delta_{CH||}$), 984 w, 891 w, 808 m, 766 s ($\delta_{CH\perp}$), 666 m (sh), 613 m, 581 m cm^{-1}. Underlined stretches belong to the characteristic set of four vibrational peaks for complexes containing {Np^{III}(Cp)$_3$} unit. Radiological concerns prohibited combustion-based evaluation of the C and H content of **KNpCp$_4$**.

Reaction of [Np^{III}(Cp')$_3$] with K: [K(2.2.2cryptand)][Np^{II}(Cp')$_3$] KNpCp'$_3$

A glass column (6 mm I.D. × 80 mm L) equipped with fritted glass disc (10-16 µm porosity) and solvent reservoir (5 mL) was fitted a straight-through PTFE-spindle valve (6 mm I.D.) and surrounding 'beaker-type' reservoir for liquid-cooling was fitted on top of a 25-mL Schlenk receiver flask. The apparatuses interior surfaces were silanized (exposure to dichloromethylsilane vapour) and heated *in vacuo* (160 °C, 6 × 10^{-4} mbar, 2 h) prior to use. The column was closely packed with large excess of KC$_8$ (H = 70 mm) and secured with a glass fibre plug, also previously silanized. For the experiment both column and receiver vessels were precooled to -35 °C with methanol/dry-ice baths under argon. A brownish-green solution of **NpCp'$_3$** (88.1 mg, 0.136 mmol), and 2.2.2-cryptand (51.1 mg, 0.136 mmol) in 1:1 THF/Et$_2$O mixture (4.0 mL) was passed through the KC$_8$ bed with a slight-overpressure of Ar. The brownish-green solution become intensely coloured, dark brown/black solution upon passage through the column and the Schlenk containing the solution was capped and cooled to -78 °C for 1 h, affording small shiny black crystals. The mother liquor was decanted and the crystals of the target [K(2.2.2cryptand)][Np^{II}(Cp')$_3$] **KNpCp'$_3$** rinsed with cold Et$_2$O (-30 °C, 2 × 2 mL). Batches of crystals were immersed in perfluoroalkyl ether oil and suitable crystals were selected for mounting inside Lindemann glass capillaries, which were manipulated through the process in a way to avoid outer surface contamination. The isolated tube was varnished by immersion in the cold (-10°C) solution of polycarbonate in dichloromethane and transferred to the goniometer head and crystals analysed by single crystal X-ray diffractometry, although only weak diffraction was observed.

Remarks: (i) Solutions of **KNpCp'$_3$** in DME are thermally unstable above approx. -10 ºC, changing suddenly in colour to pale olive-green, precluding further solution analyses under the required radiological protection regimes. (ii) Isolated crystalline samples of **KNpCp'$_3$** begin to deteriorate at room temperature within 1 minute of contact with the degassed perfluoroalkyl ether oil, with the gradual deposition of an off-white crystalline solid, assumed to be liberated 2.2.2-cryptand observed.

NMR Spectroscopic Data

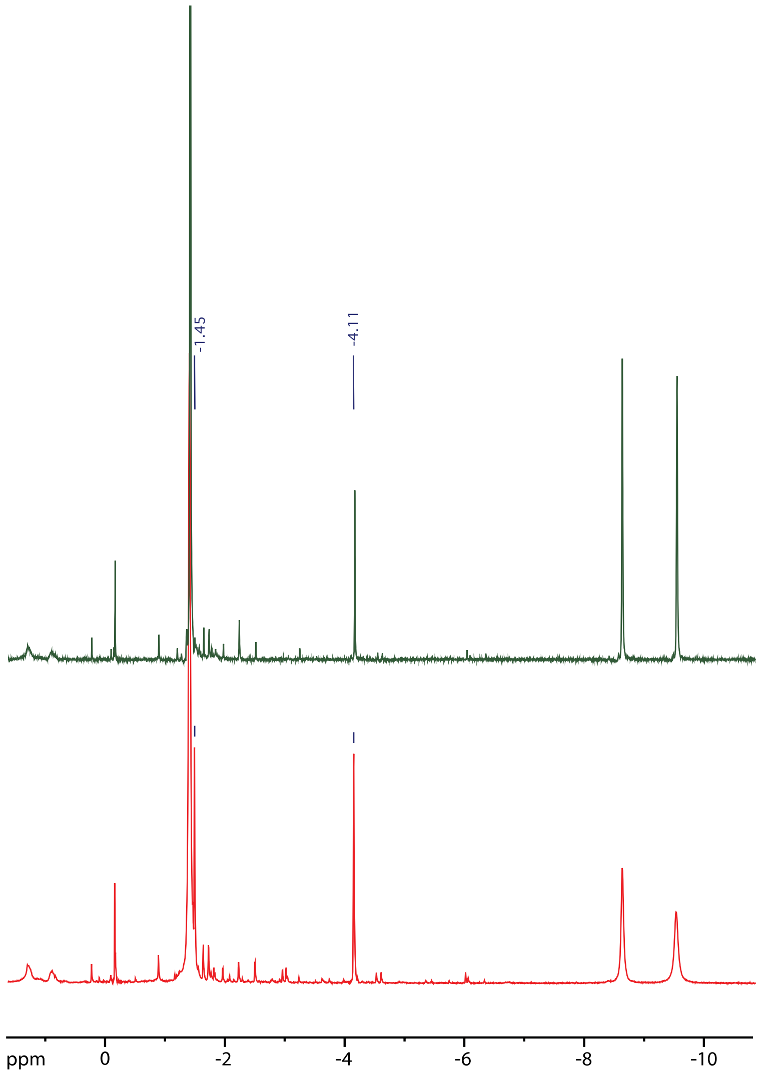

Figure SI1. Sections of the ^{1}H NMR spectra of **NpCp'$_3$** in toluene-d_8 after loading the 7.6 mM solution into the 4 mm O.D. fluoropolymer NMR tube liner (*ca.* 10 min, green trace) and followed by storage at room temperature 105 min. (red trace). The highest intensity resonance at -1.38 ppm was cut off for clarity and signal intensities were normalized against the residual protio-solvent signal at 2.08 ppm, showing the gradual decomposition of **NpCp'$_3$** and concomitant formation of the two paramagnetically shifted resonances of presumably fluorine-containing neptunium species (labelled with chemical shift).

Crystallographic Data

Crystallographic Experimental Data

Samples were immersed in perfluoroalkylether oil (1800 cSt, degassed) and suitable crystals were selected for mounting inside Lindemann glass capillaries, which were manipulated throughout the process in a way to avoid outer surface contamination. The tube was sealed and varnished with a solution of polycarbonate in dichloromethane and transferred to the goniometer head. Single crystal XRD measurements of [Np(Cp)$_3$], [Np(Cp)$_3$(NCMe)$_2$], [Np(CpTMS)$_3$], K[Np(Cp)$_4$] were performed on a Bruker Apex II Quazar diffractometer collecting two or more spheres of data. [8] Single crystal XRD measurements of [Np(Cp)$_4$] were performed on a Siemens SMART diffractometer collecting two spheres of data. [9] The data were integrated with SAINT [8, 9] corrected to Lorentz and polarisation effects and an empirical adsorption correction with SADABS [10] was applied. The structures were solved by direct methods and refined to the optimum R_1 value with SHELXL2013. [11]

Hydrogen atom parameters were constrained. Visualization and rendering of the structures was performed with OLEX2.[12] The crystallographic data have been deposited at The Cambridge Crystallographic Data Centre with the reference CCDC numbers 1524162-1524166. These data can be obtained free of charge from the CCDC *via* http://www.ccdc.cam.ac.uk/data_request/cif.
All formulas and related values given in the tables refer to the crystallographic independent unit of the elementary cell.

[Np(Cp)$_4$], **NpCp$_4$**

Empirical formula	$C_{2.50}$ $H_{2.50}$ $Np_{0.12}$	
Formula weight	62.17	
Temperature	200(2) K	
Wavelength	0.71073 Å	
Crystal system	Tetragonal	
Space group	I-42m	
Unit cell dimensions	a = 8.5980(1) Å	α= 90°
	b = 8.5980(1) Å	β= 90°
	c = 10.5541(2) Å	γ = 90°
Volume	780.22(2) Å^3	
Z	16	
Density (calculated)	2.117 Mg/m^3	
Absorption coefficient	6.650 mm^{-1}	
F(000)	466	
Crystal size	0.4 x 0.3 x 0.15 mm^3	
Theta range for data collection	3.056 to 28.230°	
Index ranges	-11<=h<=10, -11<=k<=11, -13<=l<=14	
Reflections collected	4046	
Independent reflections	530 [R(int) = 0.0232]	
Completeness to theta = 25.000°	99.6 %	
Refinement method	Full-matrix least-squares on F^2	
Data / restraints / parameters	530 / 0 / 49	
Goodness-of-fit on F^2	1.116	
Final R indices [I>2sigma(I)]	R1 = 0.0083, wR2 = 0.0187	
R indices (all data)	R1 = 0.0083, wR2 = 0.0187	
Absolute structure parameter	0.12(2)	
Extinction coefficient	n/a	
Largest diff. peak and hole	0.260 and -0.165 e.Å^{-3}	

[Np(Cp)$_3$], **NpCp$_3$**

Empirical formula	C_{15} H_{15} Np	
Formula weight	432.27	
Temperature	100(2) K	
Wavelength	0.71073 Å	
Crystal system	Orthorhombic	
Space group	Cmc2$_1$	
Unit cell dimensions	a = 14.1208(6) Å	α= 90°.
	b = 8.7037(6) Å	β= 90°.
	c = 9.5917(5) Å	γ = 90°.
Volume	1178.85(11) Å^3	
Z	4	
Density (calculated)	2.436 Mg/m^3	
Absorption coefficient	8.782 mm^{-1}	
F(000)	792	
Crystal size	0.133 x 0.103 x 0.074 mm^3	
Theta range for data collection	2.749 to 28.355°	
Index ranges	-18<=h<=18, -11<=k<=11, -12<=l<=12	

Reflections collected	10413
Independent reflections	1483 [R(int) = 0.0211]
Completeness to theta = 25.000°	99.8 %
Refinement method	Full-matrix least-squares on F^2
Data / restraints / parameters	1483 / 13 / 116
Goodness-of-fit on F^2	1.033
Final R indices [I>2sigma(I)]	R1 = 0.0141, wR2 = 0.0355
R indices (all data)	R1 = 0.0145, wR2 = 0.0359
Absolute structure parameter	0.47(7)
Extinction coefficient	n/a
Largest diff. peak and hole	1.045 and -1.123 e.Å^{-3}

[K{Np(Cp)$_4$}], **K[NpCp$_4$]**

Empirical formula	$C_{32}H_{35}K_{1.50}Np_{1.50}O_{0.50}$
Formula weight	841.75
Temperature	100(2) K
Radiation	Mo Kα (λ = 0.71073 Å)
Crystal system	Monoclinic
Space group	C_2
Unit cell dimensions	*a* = 22.6093(10) Å α = 90°
	b = 14.7125(10) Å β = 90.4150(10)°
	c = 9.0155(5) Å γ = 90°
Volume	2998.8(3) Å^3
Z	4
Density (calculated)	1.864 Mg/m^3
Absorption coefficient	5.406 mm^{-1}
F(000)	1596
Crystal size	0.044 × 0.032 × 0.024 mm^3
Theta range for data collection	1.651 to 28.568°
Index ranges	-30 ≤ h ≤ 29, -19 ≤ k ≤ 19, -12 ≤ l ≤ 11
Reflections collected	27617
Independent reflections	7071 [R(int) = 0.0474]
Completeness to theta = 25.000°	100.0 %
Absorption correction	mutli-scan method (SADABS)
Refinement method	Full-matrix least-squares on F^2
Data / restraints / parameters	7071 / 22 / 272
Goodness-of-fit on F^2	1.062
Final R indices [I>2sigma(I)]	R1 = 0.0330, wR2 = 0.0753
R indices (all data)	R1 = 0.0399, wR2 = 0.0783
Absolute structure parameter	0.000(11)
Extinction coefficient	n/a
Largest diff. peak and hole	1.304 and -0.868 e.Å^{-3}

[Np(Cp)$_3$(NCMe)$_2$], **NpCp$_3$N$_2$**

Empirical formula	$C_{19}H_{21}N_2Np$
Formula weight	514.38
Temperature	100(2) K
Wavelength	0.71073 Å
Crystal system	Orthorhombic
Space group	Pbcn
Unit cell dimensions	a = 13.6966(15) Å α= 90°.

	b = 8.3864(9) Å	β= 90°.
	c = 14.6562(16) Å	γ = 90°.
Volume	1683.5(3) Å^3	
Z	4	
Density (calculated)	2.029 Mg/m^3	
Absorption coefficient	6.171 mm^{-1}	
F(000)	968	
Crystal size	0.035 x 0.028 x 0.009 mm^3	
Theta range for data collection	2.780 to 28.418°	
Index ranges	-18<=h<=18, -11<=k<=11, -19<=l<=19	
Reflections collected	27124	
Independent reflections	2048 [R(int) = 0.0650]	
Completeness to theta = 25.000°	99.9 %	
Refinement method	Full-matrix least-squares on F^2	
Data / restraints / parameters	2048 / 0 / 93	
Goodness-of-fit on F^2	1.053	
Final R indices [I>2sigma(I)]	R1 = 0.0237, wR2 = 0.0374	
R indices (all data)	R1 = 0.0562, wR2 = 0.0431	
Extinction coefficient	n/a	
Largest diff. peak and hole	0.724 and -1.091 e.Å^{-3}	

[Np(Cp')$_3$], **NpCp'$_3$**

Empirical formula	$C_{24}H_{39}NpSi_3$	
Formula weight	648.82	
Temperature	100(2) K	
Radiation	Mo Kα (λ = 0.71073 Å)	
Crystal system	Orthorhombic	
Space group	Pbca	
Unit cell dimensions	*a* = 8.2980(6) Å	α = 90°
	b = 22.1664(17) Å	β = 90°
	c = 28.917(2) Å	γ = 90°
Volume	5318.8(7) Å^3	
Z	8	
Density (calculated)	1.620 Mg/m^3	
Absorption coefficient	4.051 mm^{-1}	
F(000)	2544	
Crystal size	0.075 × 0.025 × 0.012 mm^3	
Theta range for data collection	1.408 to 28.359°	
Index ranges	-11 ≤ h ≤ 9, -28 ≤ k ≤ 22, -38 ≤ l ≤ 37	
Reflections collected	42491	
Independent reflections	6360 [R(int) = 0.0919]	
Completeness to theta = 25.000°	100.0 %	
Absorption correction	multi-scan method (SADABS)	
Refinement method	Full-matrix least-squares on F^2	
Data / restraints / parameters	6360 / 0 / 268	
Goodness-of-fit on F^2	1.027	
Final R indices [I>2sigma(I)]	R1 = 0.0393, wR2 = 0.0490	
R indices (all data)	R1 = 0.0866, wR2 = 0.0567	
Extinction coefficient	n/a	
Largest diff. peak and hole	1.200 and -1.276 e.Å^{-3}	

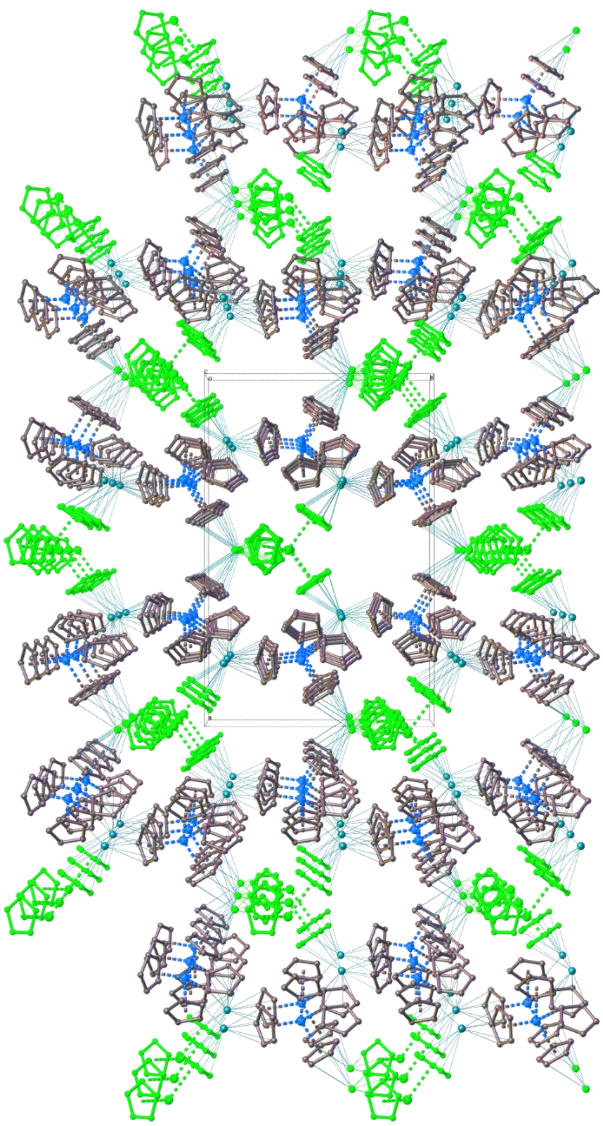

Figure SI 2. Representation of the crystal packing showing the channel structure of [K{Np(Cp)$_4$}] down the *c*-axis. All hydrogen atoms and lattice solvent molecules were omitted for clarity. The K[Np(η^5-Cp)$_4$] substructure is coloured green for emphasis; Np (blue), K (turquoise) and C (grey).

References

1. A. Streitwieser, U. Mueller-Westerhoff, F. Mares, C. B. Grant, D. G. Morrell, T. J. Marks and S. S. Miller, *Journal*, 1979, **19**, 149-154.
2. J. A. Porter, *Industrial & Engineering Chemistry Process Design and Development*, 1964, **3**, 289-292.
3. P. Laubereau, personal communication.
4. T. K. Panda, M. T. Gamer, P. W. Roesky, H. Yoo and D. H. Berry, *Journal*, 2014, 35-37.
5. J. K. Peterson, M. R. MacDonald, J. W. Ziller and W. J. Evans, *Organomet.*, 2013, **32**, 2625-2631.
6. F. Baumgärtner, E. O. Fischer, B. Kanellakopulos and P. Laubereau, *Angew. Chem. Int. Ed.*, 1968, **7**, 634-634.
7. E. Dornberger, R. Klenze, B. Kanellakopulos, *Inorg. Nucl. Chem. Letters*, 1978, **14**, 319-324.
8. Bruker. *APEXII, SAINT,* Bruker AXS Inc., Madison, Wisconsin, USA, 2012.
9. Siemens. *SMART, SAINT,* Siemens Analytical X-ray Instruments Inc., Karlsruhe, GER, 1997.
10. G. M. Sheldrick, *SADABS,* University of Göttingen, Germany, 1996.
11. G. M. Sheldrick, *Acta Cryst.*, 2015, **C71**, 3-8.
12. O. V. Dolomanov, L. J. Bourhis, R. J. Gildea, J. A. K. Howard and H. Puschmann, *J. Appl. Crystallogr.*, 2009, **42**, 339-341.
